# Small Things in Surgery

**Published:** 1868-06

**Authors:** E. Andrews

**Affiliations:** Prof. of Principles and Practice of Surgery, Chicago Medical College


ARTICLE XX.

### SMALL THINGS IN SURGERY.

BY E. ANDREWS, M.D., Prof. of Principles and Practice of Surgery,
Chicago Medical College.

It often happens that little matters in surgical practice are less readily managed by the practitioner than great ones, and that hints on such points are extremely useful. The following remarks are offered, in the hope that they may be practically advantageous to the reader:

*To Remove a Ligature which is Slow in Coming Away from a Vessel.*—Cut a smooth, round stick or piece of bougie long enough to rest across the wound. Tie the end of the ligature to the middle of the stick, or, if the thread is not long enough, tie another piece to the end of it. Turn the stick around like the handle of a gimlet, till the thread is twisted tight; and let it be turned a little more two or three times a-day. This will soon bring the ligature away.

*To Tap the Abdomen without a Trochar.*—If the surgeon finds himself called upon to perform paracentesis abdominis, and has not his trochar at hand, he may accomplish it perfectly by taking a scalpel or lancet and puncturing the linea alba, and then inserting a clean catheter. If there is no catheter at hand, a smooth wire bent in the form of a staple may be used, pushing the folded end into the wound and then turning it across the incision to keep it open. If the scalpel or lancet is sharp, care must be exercised not to thrust it too deeply.

*To Place a Seton in a Hydrocele with but One Puncture.*—Insert a trochar and withdraw the stilette; then, with a probe, thrust a tape through the tube in folds, coiling them up in the sac, in the manner recommended by Prof. Byford for certain uterine tumors; then withdraw the canula, leaving one end of the tape hanging from the wound. If no trochar is at hand, make a free incision into the sac and thrust in the tape by the side of the knife before withdrawing the blade.

*To Reduce a Hernia without the ordinary Taxis.*—The European surgeons are practising such reductions by winding layer after layer of elastic bandage upon the scrotum, until the tension of the rubber forces the gut back into the abdomen. These bandages are sold by Degenhardt and Bliss & Sharp, of this city.

*Caution in Extirpation of Piles.*—There is hardly any operation in surgery so certain of success, and at the same time so useful, as the removal of hemorrhoids, whether by the ligature, the ecraseur, or the ecraseur-forceps. One necessary precaution is, however, left without mention by many of the books. When piles occupy nearly the whole verge of the anus, they should not be severed close to their bases, because the contraction of the cicatrix induces stricture of the anus. It is sufficient if they are removed at a point about half way between their bases and their summits; the contraction of the scars causes the stumps to atrophy, and at the same time leaves integument enough to form a free verge to the anus.

*The Ecraseur-Forceps in the Removal of Tumors.*—In a former number of the EXAMINER I gave a figure and description of this instrument, which I had devised as a substitute for the chain ecraseur. I have recently applied it not only to hemorrhoids, but to the removal of cancer of the tongue. The facility with which a portion of the tongue may be removed by it without troublesome hemorrhage is very gratifying. It is equally applicable to almost all other cases where the chain ecraseur has usually been required.

*Substitute for Bullet Forceps.*—The small instrument shaped like polypus forceps in the pocket-cases makes an excellent bullet forceps for many pistol wounds. Those of them which have good points can be used to bite out a chip of lead, and thus enable the surgeon to distinguish a fractured bone from the bullet.

*Probe for Detecting Bullets.*—If a piece of the stem of a clay pipe be slipped upon the end of a probe, which has been filed down for the purpose, and then smoothed with the file, it forms a more delicate test of lead than the famous Nélaton's probe, which was used on Garibaldi. It is introduced until it touches the suspected object, and then is twirled around in contact with it. If it touched lead, the trace of it will be found upon the clay. This probe was invented by an army surgeon in Wisconsin, whose name I have forgotten.

